# The Haematological Malignancy Research Network (HMRN): a new information strategy for population based epidemiology and health service research

**DOI:** 10.1111/j.1365-2141.2009.08010.x

**Published:** 2009-12-01

**Authors:** Alexandra Smith, Eve Roman, Debra Howell, Richard Jones, Russell Patmore, Andrew Jack, on behalf of the Haematological Malignancy Research Network (members listed in Appendix 1)

**Affiliations:** 1 Epidemiology & Genetics Unit, Department of Health Sciences, University of YorkYork; 2 Haematological Malignancy Diagnostic Service, St James's Institute of Oncology, Bexley Wing, St James's University HospitalLeeds; 3 Queens Centre for Oncology & Haematology, Castle Hill HospitalHull, UK

**Keywords:** diagnostic haematology, epidemiology, leukaemia, lymphoma, myeloma

## Abstract

The Haematological Malignancy Research Network (HMRN) was established in 2004 to provide robust generalizable data to inform clinical practice and research. It comprises an ongoing population-based cohort of patients newly diagnosed by a single integrated haematopathology laboratory in two adjacent UK Cancer Networks (population 3·6 million). With an emphasis on primary-source data, prognostic factors, sequential treatment/response history, and socio-demographic details are recorded to clinical trial standards. Data on 8131 patients diagnosed over the 4 years 2004–08 are examined here using the latest World Health Organization classification. HMRN captures all diagnoses (adult and paediatric) and the diagnostic age ranged from 4 weeks to 99 years (median 70·4 years). In line with published estimates, first-line clinical trial entry varied widely by disease subtype and age, falling from 59·5% in those aged <15 years to 1·9% in those aged over 75 years – underscoring the need for contextual population-based treatment and response data of the type collected by HMRN. The critical importance of incorporating molecular and prognostic markers into comparative survival analyses is illustrated with reference to diffuse-large B-cell lymphoma, acute myeloid leukaemia and myeloma. With respect to aetiology, several descriptive factors are highlighted and discussed, including the unexplained male predominance evident for most subtypes across all ages.

## Background

Haematological malignancies are common, being the fourth most frequently diagnosed cancer in both males and females in the economically developed regions of the world (Ferlay *et al*, 2004; Jemal *et al*, 2008; Westlake, 2008). There are over 60 recognized disease subtypes, which differ widely in clinical presentation, treatment requirements and prognosis (World Health Organization [WHO], 2008). Key to the delivery and management of high-quality cancer services for this diverse patient group is a successful information strategy, with access to reliable, detailed and relevant data on disease frequency and outcome (Jensen *et al*, 1991; National Institute for Clinical Excellence [NICE], 2003; Department of Health, 2007). Equally, it is critically important to accurately describe and understand underlying disease patterns in order to originate and test hypotheses about pathogenesis (Boyle & Levin, 2008).

Globally, cancer information gathering and dissemination is sustained by national programmes, such as the Surveillance, Epidemiology and End Results (SEER) Program in the United States (US) (http://www.seer.cancer.gov) and the National Cancer Research Institute (NCRI) in the United Kingdom (UK) (http://www.ncri.org.uk), as well as international organizations, such as the WHO International Agency for Research on Cancer (http://www.iarc). The success of these cancer information systems should be judged not only by the utility of their data, but also by the confidence that the end-users have in the accuracy and analytical methods employed. Whilst meeting these exacting requirements is challenging for any cancer, it is particularly problematic for haematological malignancies where information gathering and dissemination has long been acknowledged as a major problem. These concerns were recently summarized by EUROCARE 4 in their statement that ‘the evolving classification and poor standardization of data collected on hematological malignancies vitiate the comparisons of disease incidence and survival over time and across regions’(Sant *et al*, 2009).

**Fig 1:**
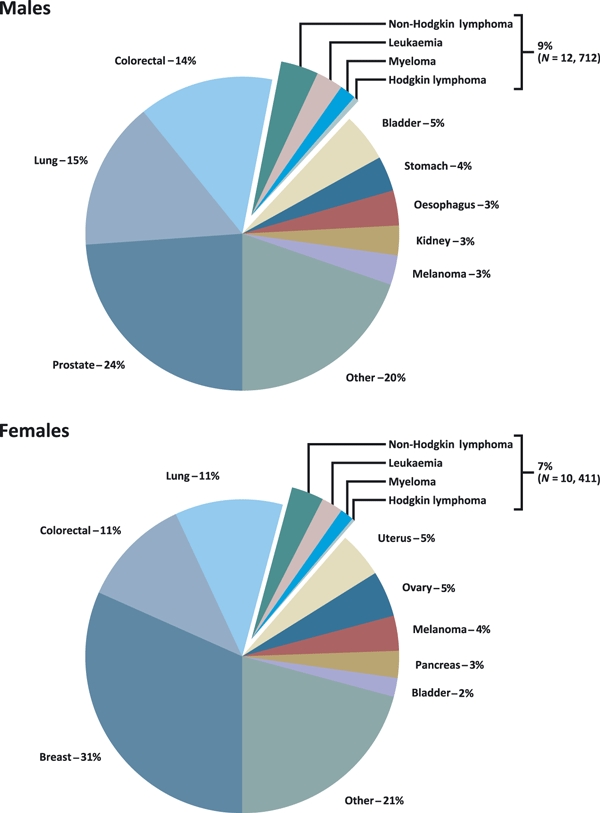
Cancer registrations United Kingdom, 2005.

A primary requirement of any successful cancer information strategy is the accurate estimation of disease burden. For haematological malignancies, two major issues need to be addressed in order to begin to produce useful data – complete unbiased ascertainment and accurate capture of detailed diagnostic data. Traditionally, descriptive information is reported in the four broad categories shown in Fig 1, which summarizes data from the UK. This practice stems from the gradual recognition of clinical entities in the latter half of the nineteenth century, and originated long before there were any effective treatments or real understanding of the relationship between haematological malignancy, the normal bone marrow and immune system, and before anything was known about the cellular and genetic basis of malignant transformation. However, the continued application of such broad categorizations severely limits the use of cancer registration data in epidemiological studies, and the high level of clinical diversity among the subtypes contained within each of the traditional groupings means that data presented in this way are of little value for health service planning and making valid comparisons of outcome (NICE, 2003; Sant *et al*, 2009).

Critically for descriptive epidemiology, the classification of haematological malignancy has changed markedly over recent decades, and will continue to do so as innovative diagnostic methods and techniques are developed (WHO, 2001, 2008). In 2001 the WHO produced, for the first time, a consensus classification that defined disease entities in terms of immunophenotype, genetic abnormalities and clinical features. However, although this classification was adopted into clinical practice almost uniformly around the world, it did not have an immediate effect on cancer registration practice, where lack of consistency in the depth and detail of pathological diagnosis means that population-based data continues to be reported in broad disease categories (Ferlay *et al*, 2004; Verdecchia *et al*, 2007; Jemal *et al*, 2008; Westlake, 2008; National Cancer Intelligence Network, 2009a; Rachet *et al*, 2009). This is largely a reflection of the fact that unlike many other cancers, haematological neoplasms are diagnosed using multiple parameters including a combination of histology, cytology, immunophenotyping, cytogenetics, imaging and clinical data. This range and depth of data is difficult for cancer registries to access systematically, forming a barrier both to the complete ascertainment and to the collection of diagnostic data at the level of detail required to implement the latest WHO classification (WHO, 2001, 2008). Furthermore, in practice, even within some of the best defined WHO categories there is a need to qualify the final diagnosis even further using additional clinical and biological prognostic factors before valid outcome comparisons can be made (The International Non-Hodgkin's Lymphoma Prognostic Factors Project, 1993; Hasenclever & Diehl, 1998; Vasconcelos *et al*, 2003; Weltermann *et al*, 2004; Buske *et al*, 2006; Sehn *et al*, 2007; Dicker *et al*, 2009).

The diagnostic complexity of haematological malignancies is mirrored by the wide diversity of treatment pathways. This diversity includes not only the intensity of treatment, but also its purpose and its duration. Whilst most cancer treatment can be categorized as potentially curative or palliative, there are increasing numbers of patients being diagnosed with more indolent forms of haematological malignancy whose life expectancy has been improved through increasingly effective therapy delivered continuously or episodically over a protracted period of time (National Institute for Clinical Excellence, 2003; O'Brien *et al*, 2003; Weber *et al*, 2007). Indeed, for many conditions, such as chronic myeloid leukaemia (CML), the prevalence of patients on active treatment is now many times greater than the annual incidence; and these treatment developments are having a major impact on the health economy (Department of Health, 2007). Longitudinal data of the type required to inform these changes are not routinely collected, but are clearly of critical importance for planning future haemato-oncology services, as well as for modelling the impact of new treatment approaches.

In response to the challenges outlined above, the Haematological Malignancy Research Network (HMRN) was established in the UK in 2004 (http://www.hmrn.org). HMRN was devised with the overarching aim of overcoming existing limitations and producing high quality functional data through the development of an innovative population-based registry. This unique venture combines the expertise of a single integrated haematopathology laboratory, an active clinical network, and data collection/analysis conducted by a specialist epidemiology unit. This paper describes the infrastructure of HMRN and discusses the use of the data, demonstrating its potential to support epidemiological research as well as health service planning and management.

## Methods

In the UK, cancer care is co-ordinated through a series of 37 area-based Cancer Networks, each covering a population between 700 000 and 3 million people. Cancer Networks are responsible for bringing together health service commissioners and providers, the voluntary sector and local authorities to deliver high quality care within the UK National Health Service (NHS). HMRN collects detailed information about all haematological malignancies diagnosed in two adjacent UK Cancer Networks; these are the Yorkshire Cancer Network and the Humber & Yorkshire Coast Cancer Network (total population 3·6 million).

Within the HMRN region, patient care is provided by a unified clinical network operating across 14 hospitals organized within five adult multi-disciplinary teams (MDTs) and a network-wide paediatric oncology service. As a matter of policy, all haematological malignancy diagnoses within the region (whether originating from the NHS or private sources and irrespective of assumed prognosis and treatment intent) are made at a single specialist haematopathology laboratory – the Haematological Malignancy Diagnostic Service (http://www.HMDS.org) – and it is from here that all HMRN patients are ascertained. HMDS, which was cited in the 2007 UK Department of Health Cancer Reform Strategy as ‘the model for delivery of complex diagnostic services’(Department of Health, 2007), provides a fully integrated diagnostic pathway in a single department, bringing together the relevant technology and expertise (including histology, cytology, immunophenotyping and molecular cytogenetics) required for the diagnosis and on-going monitoring of all haematological malignancies. A sophisticated custom-designed web database is used to handle clinical diagnoses, specimen tracking and reporting; all diagnoses, including disease transformations and progressions, are automatically coded to International Classification of Diseases for Oncology, 3rd Edition (ICD-O-3) (WHO, 2000).

Network clinical teams work to common guidelines covering investigation, treatment and follow-up. Following diagnosis a core clinical dataset is extracted for all patients from medical records at each of the 14 HMRN hospitals. Whilst the vast majority of patients are treated within haematology, records are traced across the various other disciplines and hospitals involved in patient care in order to reflect the totality of the pathway. The information collected includes demographic details, prognostic factors including imaging, and a full sequential treatment history with response and outcome recorded for all episodes. These data are acquired by a process of active collection by expert HMRN dedicated research staff, working to agreed operating procedures and data standards, with strict and continuous cross-validation within a transparent peer review process. A critically important feature of data acquisition is the emphasis on primary source information; and whilst details of disease stage at diagnosis are recorded if documented in the medical records, primary data from radiology reports, blood tests, clinical examination, and clinician summaries are also recorded, enabling embedded algorithms to automatically generate stage and prognostic scores. All details are abstracted onto structured forms (each malignancy having its own specially adapted version) and entered onto the web-based system, which integrates HMRN and HMDS data. Full details of the HMRN and copies of data abstraction forms are shown on our website (http://www.hmrn.org).

HMRN has full ethical approval and Section 60 exemption (now Section 251) to collect data for audit and research on haematological malignancy patients diagnosed within the region. For the purposes of the present analysis, population data were obtained for the HMRN region and for the UK as a whole from the 2001 census (Office for National Statistics, 2001). Incidence rates and corresponding 95% confidence intervals (CIs) were estimated using Poisson regression and survival curves by the Kaplan–Meier method. All analyses were conducted using the Stata 10 statistical software (StataCorp LP, College Station, TX, USA).

## Results

Descriptive findings are presented here for 8131 patients newly diagnosed with a haematological neoplasm between 1 September 2004 and 31 August 2008 in the HMRN region. Of these, 224 (2·8%) patients had a second haematological neoplasm diagnosed during the 4-year period either because of disease progression or transformation, or because of a concurrent diagnosis with a different cell lineage, yielding 8355 diagnoses in total. These 8355 diagnoses are distributed according to the WHO 2001 classification (WHO, 2001) in Table I. Twenty-three main groupings are shown in bold, and contributory subtypes with five or more diagnoses are also listed. In addition to frequency, information on sex (% male), age at diagnosis (median) and first line clinical trial recruitment (% of total) are also presented.

**Table I:** Diagnostic characteristics: Haematological Malignancy Research Network (HMRN), 2004–2008

Neoplasm (ICD–O3)	Total	Males (%)	Median age at diagnosis (years)	First Line treatment trial entry (%)
**All diagnoses**	**8355**	**54·9**	**70·4**	**7·2**
**Chronic myeloid leukaemia (9875/3)**	**137**	**61·3**	**59·1**	**11·0**
**Myeloproliferative neoplasms**	**822**	**41·5**	**71·4**	**0·2**
Chronic myeloproliferative neoplasm (9960/3)	750	40·1	71·2	0·3
Chronic myeloproliferative neoplasm with myelofibrosis (9961/3)	55	61·8	73·9	–
Hypereosinophilic syndrome (9964/3)	9	44·4	55·5	–
Systemic mastocytosis (9741/3)	8	25·0	62·5	–
**Chronic myelomonocytic leukaemia (9945/3)**	**76**	**63·2**	**75·6**	**–**
**Myelodysplastic syndromes**	**509**	**69·0**	**75·9**	**4·7**
Refractory anaemia with ringed sideroblasts (9982/3)	96	62·5	77·4	–
Refractory anaemia with excess blasts (9983/3)	220	68·2	75·1	9·5
Refractory cytopenia with multilineage dysplasia (9985/3)	193	73·1	75·9	–
**Acute myeloid leukaemia (AML)**	**570**	**52·1**	**68·5**	**34·4**
AML not otherwise specified (NOS) (9861/3 9895/3)	462	52·6	71·4	32·3
AML with core binding factors (9871/3 9896/3)	28	64·3	42·9	53·6
AML – probable therapy-related (9920/3)	26	38·5	71·0	7·7
AML with *MLL* (11q23) rearrangement (9897/3)	10	30·0	17·9	70·0
APML t(15;17)(q22;q11–12) (9866/3)	44	52·3	38·5	52·3
**Precursor B-lymphoblastic leukaemia (9836/3)**	**137**	**52·6**	**12·8**	**68·6**
**Monoclonal B-cell Lymphocytosis**	**385**	**53·0**	**71·6**	**–**
**Chronic lymphocytic leukaemia (9823/3)**	**835**	**62·0**	**71·8**	**0·6**
**Marginal zone lymphoma**	**390**	**54·4**	**71·9**	**3·1**
Systemic marginal zone lymphoma (9699/3)	296	56·8	72·2	4·1
Extranodal marginal zone lymphoma (9699/3)	94	46·8	68·9	–
**Hairy cell leukaemia (9940/3)**	**48**	**75·0**	**68·0**	**–**
**Monoclonal gammopathy of undetermined significance (9765/1)**	**892**	**56·4**	**72·2**	**–**
**Plasma cell myeloma (9732/3)**	**876**	**57·0**	**72·8**	**13·7**
**Plasmacytoma (9731/3, 9734/3)**	**68**	**64·7**	**68·8**	**13·2**
**Follicular lymphoma (9690/3, 9698/3)**	**446**	**46·2**	**64·1**	**3·8**
**Mantle cell lymphoma (9673/3)**	**100**	**62·0**	**74·3**	**8·0**
**Diffuse large B-cell lymphoma (9680/3)**	**1098**	**52·6**	**70·0**	**4·5**
Diffuse large B-cell lymphoma (9680/3)	1085	52·6	70·2	4·5
Mediastinal large B-cell lymphoma (9679/3)	13	53·8	31·7	–
**Burkitt lymphoma (9687/3)**	**54**	**79·6**	**48·2**	**7·4**
**Lymphoproliferative disorders NOS (9823/3, 9591/3)**	**263**	**52·9**	**76·8**	**–**
**Precursor T-lymphoblastic leukaemia (9837/3)**	**43**	**65·1**	**17·5**	**55·8**
**T-cell leukaemia**	**50**	**48·0**	**75·0**	**2·0**
T-cell or natural killer cell large granular lymphocytosis (9831/3)	40	52·5	72·7	2·5
T-cell prolymphocytic leukaemia (9834/3)	10	30·0	78·3	–
**T-cell lymphoma**	**156**	**57·7**	**64·3**	**0·6**
Peripheral T-cell lymphoma – common; unspecified (9702/3)	49	63·3	68·6	–
Mycosis fungoides (9700/3)	28	71·4	64·7	–
Primary cutaneous CD30 positive T-cell (9718/3)	23	43·5	52·3	–
Anaplastic large cell lymphoma of T/null type (9714/3)	18	66·7	55·6	5·6
Angioimmunoblastic T-cell lymphoma (9705/3)	16	31·3	74·0	–
Enteropathy-type T-cell lymphoma (9717/3)	10	50·0	62·8	–
Extranodal NK/T-cell lymphoma, nasal type (9719/3)	9	44·4	61·6	–
**Lymphocyte predominant nodular Hodgkin lymphoma (9659/3)**	**40**	**72·5**	**43·6**	**–**
**Classical Hodgkin Lymphoma**	**360**	**51·1**	**42·1**	**5·8**
Nodular sclerosis classical Hodgkin lymphoma (9663/3)	262	48·9	37·0	5·7
Mixed cellularity classical Hodgkin lymphoma (9652/3)	91	54·9	60·0	5·5
Lymphocyte-rich classical Hodgkin lymphoma (9651/3)	7	85·7	56·6	14·3

**Fig 2:**
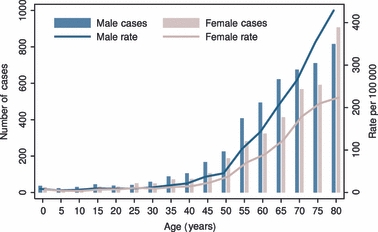
Age and sex distribution: Haematological Malignancy Research Network (HMRN), 2004–2008.

**Fig 3:**
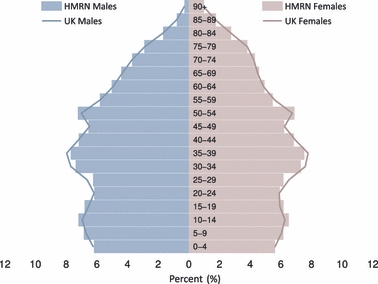
Population age and sex structure of the Haematological Malignancy Research Network (HMRN) region (bars) compared to the UK as a whole (lines), 2001.

Whist a haematological malignancy can occur at any age, as with most other cancers, the likelihood of diagnosis increased markedly with increasing age (Fig 2). The median age at diagnosis within HMRN over the 4 years 2004–08 was 70·4 years for all haematological neoplasms combined, with a range of 4 weeks to 99 years. Age-specific male rates were generally higher than female rates (lines in Fig 2), the divergence between the two becoming progressively more marked over the age of 50 years. There was clearly a pronounced male excess across the majority of myeloid and lymphoid subtypes, but despite this, more women than men were diagnosed over the age of 80 years (bars in Fig 2). This apparent discrepancy arose because more women than men survive to reach old age, as can be seen from Fig 3 which shows the age and sex structure of HMRN's population (bars) as well as that of the UK as a whole (lines).

With a combined population of 3·6 million it is, perhaps, not surprising that HMRN's regional structure mirrored that of the UK as a whole in terms of age and sex (Fig 3). Average annual incidence rates for the 23 main groups and expected annual frequencies for the UK as a whole, estimated by applying HMRN sex- and age- (5-year age strata) specific rates to equivalent UK sex- and age-specific population strata, are presented in Table II. A direct comparison with published figures could not be made as national cancer registrations are not coded to ICD-O-3, and not all of the categories shown in Table II were uniformly compiled (myeloproliferative neoplasms and myelodysplastic syndromes, for example). This is one of the reasons why, for both males and females, the overall estimated levels based on HMRN rates are almost 50% higher than the 2005 UK cancer registration frequencies presented in Fig 1. More detailed downloadable user-defined breakdowns by subtype, age and sex can be obtained from our website (http://www.hmrn.org).

**Table II:** Average annual rates (95% confidence intervals) per 100 000 within the HMRN region 2004–2008, and estimated annual numbers for the UK as a whole*

	Total		Males		Females	
			
Neoplasm	Rate (95% CI)	Estimated UK annual cases	Rate (95% CI)	Estimated UK annual cases	Rate (95% CI)	Estimated UK annual cases
All diagnoses	58·5 (57·3–59·8)	33 376	66·3 (64·4–68·3)	18 602	51·2 (49·6–52·9)	14 774
Chronic myeloid leukaemia	1·0 (0·8–1·1)	566	1·2 (1·0–1·5)	350	0·7 (0·5–0·9)	216
Myeloproliferative neoplasm	5·8 (5·4–6·2)	3286	4·9 (4·4–5·5)	1391	6·6 (6·0–7·2)	1895
Chronic myelomonocytic leukaemia	0·5 (0·4–0·7)	297	0·7 (0·5–0·9)	192	0·4 (0·3–0·6)	105
Myelodysplastic syndromes	3·6 (3·3–3·9)	2018	5·1 (4·6–5·7)	1417	2·2 (1·8–2·5)	602
Acute myeloid leukaemia	4·0 (3·7–4·3)	2274	4·3 (3·8–4·8)	1202	3·7 (3·3–4·2)	1073
Precursor B-lymphoblastic leukaemia	1·0 (0·8–1·1)	560	1·0 (0·8–1·3)	291	0·9 (0·7–1·1)	269
Monoclonal B-cell lymphocytosis	2·7 (2·4–3·0)	1551	3·0 (2·6–3·4)	835	2·5 (2·1–2·8)	716
Chronic lymphocytic leukaemia	5·9 (5·5–6·3)	3311	7·5 (6·9–8·2)	2094	4·3 (3·8–4·8)	1218
Marginal zone lymphoma	2·7 (2·5–3·0)	1552	3·1 (2·7–3·5)	858	2·4 (2·1–2·8)	695
Hairy cell leukaemia	0·3 (0·3–0·5)	203	0·5 (0·4–0·7)	154	0·2 (0·1–0·3)	49
Monoclonal gammopathy of uncertain significance	6·2 (5·8–6·7)	3544	7·3 (6·6–7·9)	2023	5·3 (4·8–5·8)	1521
Plasma cell myeloma	6·1 (5·7–6·6)	3459	7·2 (6·6–7·8)	1998	5·2 (4·6–5·7)	1461
Plasmacytoma	0·5 (0·4–0·6)	277	0·6 (0·5–0·9)	180	0·3 (0·2–0·5)	97
Follicular lymphoma	3·1 (2·8–3·4)	1800	3·0 (2·6–3·4)	840	3·3 (2·9–3·7)	960
Mantle cell lymphoma	0·7 (0·6–0·9)	395	0·9 (0·7–1·2)	249	0·5 (0·4–0·7)	146
Diffuse large B-cell lymphoma	7·7 (7·3–8·2)	4371	8·3 (7·7–9·0)	2326	7·1 (6·5–7·7)	2045
Burkitt lymphoma	0·4 (0·3–0·5)	222	0·6 (0·5–0·8)	176	0·2 (0·1–0·3)	46
Lymphoproliferative disorder NOS†	1·8 (1·6–2·1)	1025	2·0 (1·7–2·4)	557	1·7 (1·4–2·0)	468
Precursor T-lymphoblastic leukaemia	0·3 (0·2–0·4)	173	0·4 (0·3–0·6)	113	0·2 (0·1–0·3)	60
T-cell leukaemia	0·4 (0·3–0·5)	204	0·4 (0·2–0·5)	100	0·4 (0·2–0·5)	104
T-cell lymphoma	1·1 (0·9–1·3)	639	1·3 (1·1–1·6)	372	0·9 (0·7–1·2)	267
Lymphocyte predominant nodular Hodgkin	0·3 (0·2–0·4)	185	0·4 (0·3–0·6)	134	0·2 (0·1–0·3)	50
Classical Hodgkin lymphoma	2·5 (2·3–2·8)	1463	2·7 (2·3–3·1)	752	2·4 (2·1–2·8)	711

* Estimated by applying HMRN sex and age (5-year strata) specific rates to equivalent UK strata (Census Dissemination Unit & Office for National Statistics, 2009).

† Not otherwise specified.

**Fig 4:**
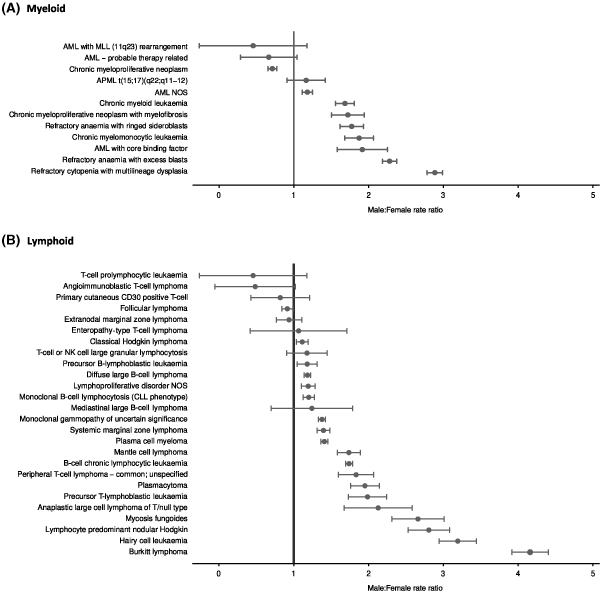
Sex rate ratios: Haematological Malignancy Research Network (HMRN), 2004–2008.

The sex-specific incidence rate ratios (male rate/female rate) together with their standard errors are shown separately for myeloid and lymphoid subtypes with 10 or more diagnoses in Fig 4, which is ordered according to the magnitude of the rate ratio. As might be expected, some related conditions had similar sex rate ratios, that of monoclonal gammopathy of undetermined significance (MGUS) and myeloma, for example, being identical (1·4; 95% CI, 1·2–1·6). Whereas for others, such as chronic lymphocytic leukaemia (CLL, 1·7; 95% CI, 1·5–2·0) and monoclonal B-cell lymphocytosis (MBL, 1·2; 95% CI, 1·0–1·5) there were differences. Variations were also evident within some of the main diagnostic categories. For acute myeloid leukaemia (AML), for example, the overall rate ratio was 1·1, but this ranged from 0·7 for therapy-related AML to 1·9 for AML with core binding factor. Likewise T-cell lymphomas, with an overall rate ratio of 1·4, ranged from for 0·5 for angioimmunoblastic T-cell lymphoma to 2·1 for anaplastic large cell lymphoma of T/null type.

**Fig 5:**
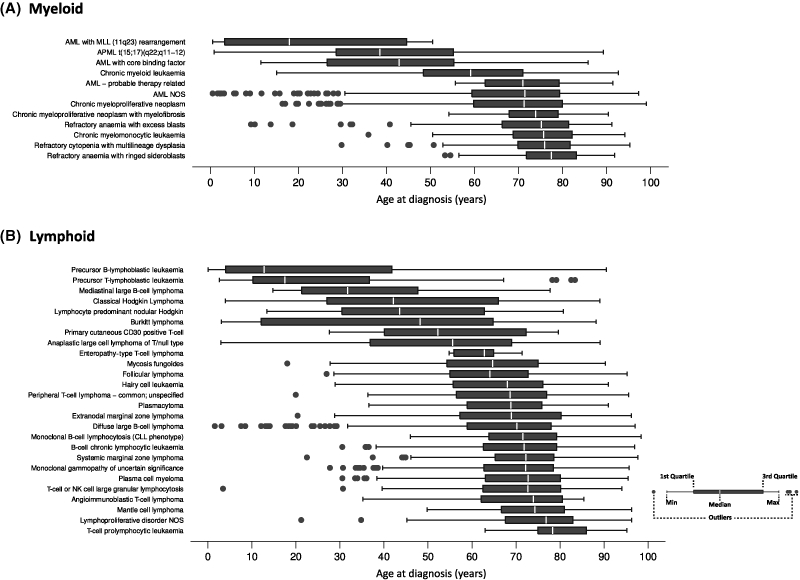
Age at diagnosis distributions: Haematological Malignancy Research Network (HMRN), 2004–2008.

Box and whisker summary age plots broadly arranged according to the magnitude of the median ages given in the third column of Table I are shown separately for myeloid and lymphoid subtypes with 10 or more diagnoses in Fig 5. Among myeloid neoplasms, AML spanned the entire age range. However, this concealed distinct patterns associated with genetically defined subtypes. For example, the median age of patients with 11q23 rearrangements was 17·9 years, demonstrating that this largely paediatric malignancy nevertheless occurs sporadically up to the age of 50 years. At the other end of the age spectrum, therapy-related AML had a median age of 71 years and was not recorded in the present series of HMRN patients before the age of 55 years. A strong relationship between age and subtype was also evident among lymphoid neoplasms, the median age at diagnosis ranging from 12·8 years for precursor B-lymphoblastic leukaemia through to 78·3 years for T-cell prolymphocytic leukaemia (Fig 5B). As well as contrasts, the similarity of the age distributions of closely related conditions was striking. MBL and CLL, for example, were adjacent in the plots – the median ages at diagnosis were 71·6 and 71·8 years, respectively. Likewise, MGUS and myeloma had median ages of 72·2 and 72·8 years, respectively.

**Fig 6:**
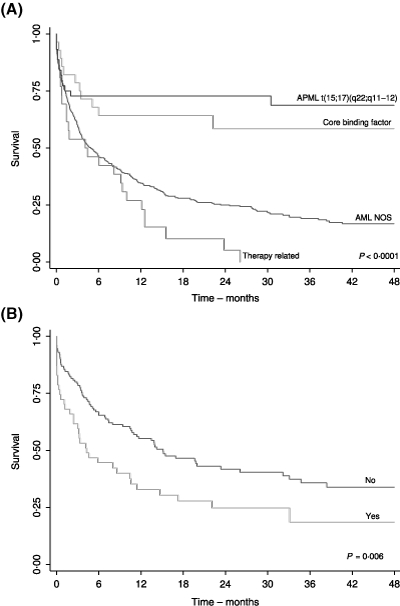
Kaplan–Meier survival estimates for (A) AML patients by ICD-O-3 subtype and (B) for AML not otherwise specified (NOS) patients according to *FLT3* length mutation status: Haematological Malignancy Research Network (HMRN), 2004–2008.

**Fig 7:**
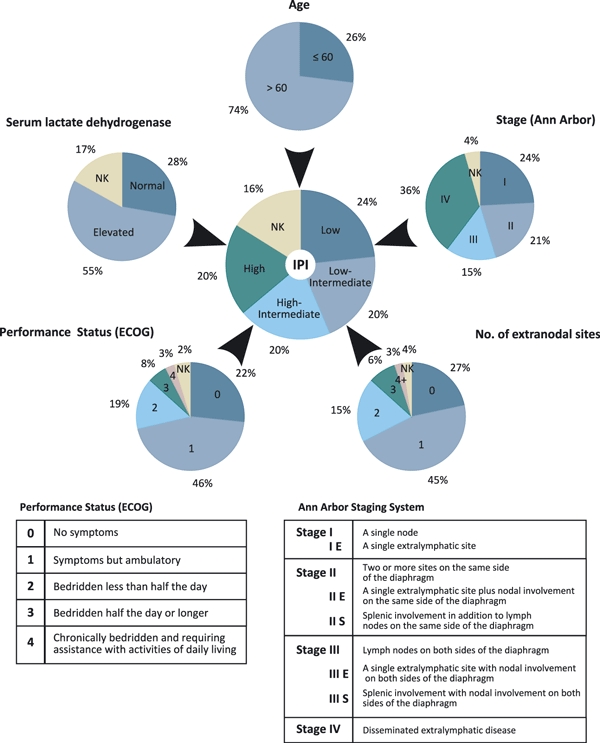
International Prognostic Index (IPI) derivation from components in diffuse large-B-cell lymphoma (DLBCL): Haematological Malignancy Research Network (HMRN), 2004–2008. (ECOG, Eastern Cooperative Oncology Group).

The importance of examining specific disease entities is further illustrated with reference to AML in Fig 6A, which plots survival for AML WHO ICD-O-3 categories with 25 or more patients. In general, prognosis for adults diagnosed with AML was recognized to be poor, but there was considerable heterogeneity by subtype with almost three-quarters of those diagnosed with acute promyelocytic leukaemia t(15;17)(q22; q11–12) surviving beyond 4 years. In addition to age and diagnostic category, additional prognostic markers also impact on survival. This is illustrated further in Fig 6B, which examines the survival of patients diagnosed with AML not otherwise specified (NOS) according to the mutation status of the tyrosine kinase receptor FLT3 – those with the mutation had a significantly poorer survival than those without it (*P* = 0·006). In the case of B-cell malignancies, clinical indices based on disease bulk and patient fitness are a well-validated method of predicting outcome. An example of these prognostic indicators for B-cell lymphomas is illustrated in Fig 7, which shows the components of the International Prognostic Index (IPI) for diffuse large B-cell lymphoma, based on 1043 patients over 18 years of age who had no prior B-cell disease. The combinations of cellular and clinical prognostic factors, the components of which vary according to disease subtype, are essential for the accurate definition of any given patient population.

The final column of Table I gives the proportion of patients entered into a clinical trial as their first line treatment, which for all haematological malignancies combined was only 7·2%, ranging from 59·5% in those under 15 years through to 1·9% in those aged 75 years or more. The highest trial entry was for precursor B-lymphoblastic leukaemia, reflecting the well recognized high levels of recruitment for this largely paediatric cancer. For many other conditions, particularly those that dominate the older age ranges, recruitment was very low, as can be seen more clearly in Table III where first line trial entry proportions are distributed by age at diagnosis and diagnostic category.

**Table III:** First-line trial recruitment distributed by age and diagnosis: Haematological Malignancy Research Network (HMRN), 2004–2008

			Age (years)					
			
		Overall	0–14	15–29	30–44	45–59	60–74	75+
All Diagnoses	Total	8355	158	242	451	1380	3098	3026
	Trial (%)	7·2	59·5	24·0	14·2	9·6	6·4	1·9
Precursor B-lymphoblastic leukaemia	Total	137	73	15	17	13	14	5
	Trial (%)	68·6	89·0	86·7	52·9	46·2	7·1	0
Precursor T-lymphoblastic leukaemia	Total	43	18	13	3	2	3	4
	Trial (%)	55·8	83·3	61·5	0	50·0	0	0
Acute myeloid leukaemia	Total	570	18	37	53	89	169	204
	Trial (%)	34·4	61·1	73·0	64·2	55·1	34·9	7·8
Plasma cell myeloma	Total	876	–	–	14	144	352	366
	Trial (%)	13·7	–	–	35·7	21·5	16·5	7·1
Chronic myeloid leukaemia	Total	137	–	8	19	46	42	22
	Trial (%)	11·0	–	0	13·3	17·4	11·9	0
Mantle cell lymphoma	Total	100	–	–	–	11	42	47
	Trial (%)	8·0	–	–	–	18·2	11·9	2·1
Burkitt lymphoma	Total	54	15	6	5	10	12	6
	Trial (%)	7·4	0	33·3	20	0	0	16·7
Classical Hodgkin lymphoma	Total	360	16	98	76	53	70	47
	Trial (%)	5·8	0	7·1	5·3	7·6	5·7	4·3
Diffuse large B-cell lymphoma	Total	1098	9	19	67	206	416	381
	Trial (%)	4·5	11·1	5·3	4·5	8·7	5·5	0·8
Other haematological malignancies	Total	4980	9	46	197	806	1978	1944
	Trial (%)	1·5	22·2	–	3·1	1·6	2·2	0·5

**Fig 8:**
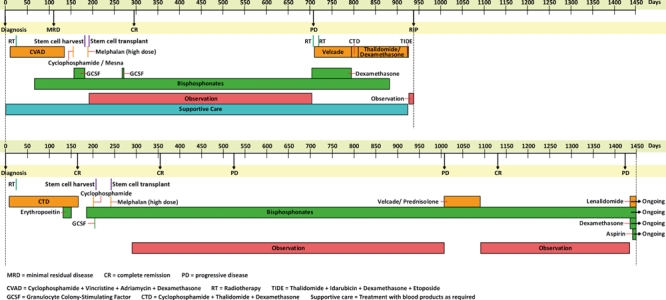
Myeloma patient pathway of two patients: Haematological Malignancy Research Network (HMRN), 2004–2008.

A key determinant of outcome and the health resource invested is the treatment the patient received throughout the entire course of their cancer pathway. With the treatment pathways of haematological malignancies having the potential to be both multifaceted and protracted, collecting and presenting these data is particularly challenging. The ability of our data to reveal this complexity is illustrated in Fig 8 which shows the pathways of two HMRN myeloma patients, one in a trial and one not in a trial. Following diagnosis, both patients were constantly monitored within the haematology department, and had multiple treatment episodes directed both at disease control and at symptom management. The first patient, whose disease followed an aggressive course, died 940 d (2·6 years) after diagnosis following intensive salvage treatment with various modalities – radiotherapy as well as multiple episodes of chemotherapy. The second patient, however, followed a more indolent course but ultimately still required multiple lines of treatment and is currently being treated with lenalidomide 4 years after diagnosis.

## Discussion

The Haematological Malignancy Research Network (HMRN) was established as a resource to support multifaceted population-based research and to provide ‘real-time’ information for monitoring and improving patient care. To achieve these aims it was necessary to design a system of data collection that attained a level of detail and completeness that is beyond the remit of conventional population-based cancer registries. The success of this programme, as illustrated by the data presented here, depended on the development of a data collection and analysis pathway that integrated the key diagnostic and clinical components in a single platform managed by an interdisciplinary team with expertise in diagnostics, clinical haematology and epidemiology.

Epidemiological reports on haematological malignancy often begin, and sometimes end, by stating that little is known about the aetiology of the condition(s) under study. The use of appropriate disease classifications is critical to the research process; hitherto, however, many studies of haematological malignancy have been hampered by the need to aggregate their data into broad groupings, either because primary source information was recorded in that way or because diagnostic standards were inconsistently applied (Ferlay *et al*, 2004; Westlake, 2008, National Cancer Intelligence Network, 2009a,b; Rachet *et al*, 2009; Sant *et al*, 2009). This study is the first to use the WHO classification (WHO, 2000, 2001, 2008) to examine the age and gender patterns of haematological neoplasms in a well defined population, and the benefits of this are immediately apparent. The analyses revealed that whilst males are far more likely than females to develop a haematological neoplasm at any age, there is considerable variation by subtype (Fig 4), which any aetiological hypothesis should seek to address. In this regard, exceptions to the generality of the male excess are also noteworthy – the excess of women with therapy-related AML, for example, being likely to reflect the use of chemo/radiotherapy in breast cancer (Martin *et al*, 2009).

The relationship between age and some lymphoma and leukaemia subtypes are also well recognized, and descriptive information is invariably published separately for children and adults. This results in data on cancers that are comparatively rare either in children or in adults often being overlooked – attention being focussed on the age group where the condition is most common. Our analysis of haematological neoplasms across the entire age-range confirmed the fact that sporadic cases of paediatric-type disease can occur in later life, and vice-versa (Fig 5). This has clear implications not only for aetiological hypotheses, but also for the delivery of care to patients with these conditions. Furthermore, the strong age distribution similarities between precursor conditions, such as MBL and MGUS and their more aggressive counterparts – respectively CLL and myeloma, provides further evidence that these conditions are part of a wider continuum (Rawstron *et al*, 2008; Landgren *et al*, 2009). Indeed, unlike other cancers, haematological malignancies are characterized by their ability to progress and transform, the longitudinal nature of these processes being captured by HMRN data acquisition procedures. In this, our first report, in-line with current cancer-registration practice, analyses were based on the number of diagnoses (*n* = 8355) rather than the number of patients (*n* = 8131). The proportion of patients with a subsequent diagnosis is currently small (2·8%) – the follow-up period reported on here being comparatively short and the findings presented being unaffected by denominator choice. Future analyses on individual conditions will factor in this complexity and be based on the number of patients, rather than their diagnoses.

A key observation within our analyses was that the estimated UK incidence of haematological neoplasms based on HMRN rates exceeds national registrations by about 50% (Table II). Whilst this may be partly due to more complete ascertainment of certain conditions, there are other contributory factors that could account for this discrepancy. It has been recognized in recent years, for example, that occult forms of CLL are not uncommon in the general population, with reports of up to 12% of adults being affected (Rawstron *et al*, 2007; Nieto *et al*, 2009). The term MBL is used when the B-cell count in the peripheral blood is less than 5 × 10^9^/l, and although this arbitrary cut-off is widely applied, MBL and CLL are part of a continuum (Rawstron *et al*, 2008). Hence, the probability that a patient with MBL or low level CLL will be diagnosed varies with both local clinical practice and local access to specialist diagnostic facilities. Many of these patients will neither require treatment nor have a reduced life expectancy – hence higher incidence often equates with better survival. Within cancer registries these patients may not be registered, be registered inconsistently, or be coded inappropriately. This explains, at least in part, the large variation in total leukaemia incidence and survival reported for the Cancer Networks in England 2001–05 (http://www.ncin.org.uk/eatlas) – the incidence ranged from 13·5 per 100 000 in Yorkshire to 7·5 per 100 000 in the adjacent Cancer Network (North of England), with corresponding 1-year survival estimates of 77·6% and 64·5%, respectively. This is highly unlikely to reflect ‘true’ underlying variation, and almost certainly represents differences in reporting and ascertainment of the many disease subtypes that comprise the ‘all leukaemias’ category. Comparable problems exist for MGUS and myeloma, other types of indolent B-lymphoproliferative disorders, as well as the lower grade forms of myelodysplasia. In any population within each of these categories, there will be a pool of undiagnosed patients, the magnitude of which will vary with local clinical practice and subsequent data recording.

Whilst individual subtype frequency comparisons between HMRN and national programmes cannot be made because their data are not coded to WHO ICD-O-3, it is nonetheless reassuring to note that HMRN's rates for clinically evident disease groupings, such as the Hodgkin lymphomas (Table II), are very similar to those of NCIN (http://www.ncin.org.uk) and SEER (http://www.seer.cancer.gov). However, even in patients with clinically acute disease, where overall levels of ascertainment are more standard, there are several issues that must be considered before valid comparisons of clinical outcome are made between treatment centres. In this regard, a further crucial step in interpreting findings is the incorporation of molecular markers. The WHO classification (WHO, 2008), for example, recognizes a number of specific AML subtypes based on cytogenetic and molecular abnormalities, each of which has well defined clinical characteristics and outcomes (Weltermann *et al*, 2004; Gale *et al*, 2008; Rau & Brown, 2009) – the survival plots presented in Fig 6 clearly show the importance of taking such markers into account. Patients with more advanced CLL provide another example where outcome and survival vary with prognostic factors such as *TP53* inactivation and immunoglobulin mutation status (Vasconcelos *et al*, 2003; Byrd *et al*, 2006; Dicker *et al*, 2009). Obviously, making comparisons between centres and individuals in the absence of data on prognostic factors could lead to erroneous conclusions being drawn. This not only has implications for local governance and research, but also for the growing number of commercial health care information providers who routinely tabulate data and rank centres.

The collection of information to a standard comparable to a clinical trial, in particular the emphasis on primary source measurements, is integral to HMRN data collection procedures. For example, for diffuse large B-cell lymphoma, follicular lymphoma and Hodgkin lymphoma, international prognostic scoring systems are widely used to stratify patients in clinical trials (Hasenclever & Diehl, 1998; Buske *et al*, 2006; Sehn *et al*, 2007) – it is, however, well recognized that such indices are not routinely recorded for non-trial patients. As illustrated in Fig 7, HMRN actively addresses this through the collection of component data from multiple primary sources, using disease-specific abstraction forms that enable embedded algorithms to accurately calculate stage and prognostic score. In the future, these data will provide the contextual framework for evaluating clinical outcomes and assessing the generalizability of clinical trial findings to the wider patient population. This is a much needed requirement in the UK because, whilst haemato-oncology has been cited as a specialty with a strong commitment to clinical trials and evidence based therapy (National Cancer Research Network, 2009a,b;), it is also recognized that, overall, as few as 5% of patients are treated in the context of a clinical trial (National Institute for Clinical Excellence, 2003). In fact, population-based figures on trial participation are rarely available for haematological malignancies, either as a whole or by subtype, largely because the totality of the patient population within the relevant disease subtype groupings is unknown – this is true both in the UK and elsewhere in the world. Indeed, whether or not a patient is entered into a clinical trial is affected by many factors, and the data presented here confirm that this process is far from random (National Institute for Clinical Excellence, 2003; Department of Health, 2007). This lack of representativeness has clear implications for the external validity of trial findings, as well as for the common tendency to extrapolate trial data for commissioning purposes (National Institute for Clinical Excellence, 2003; Murthy *et al*, 2004; Cronin *et al*, 2005; Vickers, 2008; Estey, 2009; Friedberg *et al*, 2009).

How patients are treated obviously affects outcome, and here again data for haematological malignancies are particularly variable and complex, as is illustrated in Fig 8, which shows two examples of HMRN myeloma patient pathways. This intricacy is captured within HMRN by abstracting complete treatment histories, which is essential because, although primary treatment is usually standardized, this is not so for relapsed patients who may require salvage therapy(s). In this case potential treatment options and the allocation to a particular treatment course increasingly depend on physician and patient choice – as well as national and local funding policies (Department of Health, 2007, 2008). A similar situation exists for more indolent conditions, such as CLL and follicular lymphoma, where the decision to initiate treatment and subsequent treatment options may vary with both individual patient and/or clinician choice, as well as with treatment centre. Importantly, in addition to facilitating analyses that will enable therapeutic decisions to be based on evidence of efficacy. The collection of entire treatment histories also reveals pathways that are amenable to comparative economic analysis. This will become increasingly relevant as many patients with haematological malignancies who would once have died from their disease fairly rapidly, now survive. However, unlike survivors from other forms of cancer they often require life-long treatment(s) – either continuously or episodically. This has major, but poorly defined implications for the health economy and as such will be a key area for future HMRN analyses aimed at informing the process of commissioning cancer services.

This review of the first 4 years of HMRN demonstrate that within the framework of the UK National Health Service (NHS), it is feasible to collect data to the standard required, both to inform patient care and to provide the foundation for current and future research. HMRN was initiated to serve both research and clinical needs, and as such the capture of all patients diagnosed and treated is a paramount objective. Accurately characterizing the case-mix, as we have been able to do, is critical for interpreting incidence patterns and for interpreting findings from comparative studies of clinical outcome, but at present, relevant data of this type are rarely systematically collected (Sehn *et al*, 2005). HMRN is based in two Cancer Networks, and as such reflects the infrastructure of national cancer care delivery in the UK where patients are diagnosed and treated locally (National Institute for Clinical Excellence, 2003; National Cancer Intelligence Network, 2009a). The age and sex structure of the population of 3·6 million mirrors that of the UK as a whole, and there is no reason to believe that the population-based findings are not generalizable to the country as a whole. Furthermore, in contrast to many other cancers there is little evidence to suggest that haematological disease varies systematically with factors such as social class (Smith *et al*, 2006; National Cancer Intelligence Network, 2009c), although broad variations with ethnicity have been reported (National Cancer Intelligence Network, 2009d). Issues such as these will be investigated in detail in future reports.

Haematological oncology is changing rapidly, with new approaches to treatment and diagnosis continually emerging as diverse patient pathways evolve. There are now examples where the use of refined diagnostic techniques and classifications is beginning to uncover underlying genetic factors in the pathogenesis of haematological neoplasms (Jones *et al*, 2009; Kilpivaara *et al*, 2009). For example, gene expression profiling and other techniques are now demonstrating the linkage between disease categories, such as mediastinal B-cell lymphoma and classical Hodgkin lymphoma (Rosenwald *et al*, 2003; Savage *et al*, 2003), while subdividing others, such as diffuse large B-cell lymphoma, in ways that may reflect underlying pathogenesis (Wright *et al*, 2003; Lossos *et al*, 2004; Tome *et al*, 2005; Malumbres *et al*, 2008; Alizadeh *et al*, 2009). Importantly, HMRN combines the necessary high quality population-based data collection systems and diagnostic facilities to further investigate the epidemiology of these emerging entities.

In conclusion, our study demonstrates that it is feasible to collect haematological malignancy data to the standard required to inform patient care and provide a solid foundation for research using the framework of the UK National Health Service (NHS). Indeed, HMRN's maturing data presents an increasingly valuable resource to address real questions of concern to haematologists, commissioners, health service researchers and patients. However, the wide-ranging challenges of acquiring sufficiently detailed information mean that this model would be extremely difficult to replicate across the UK as a whole. Accordingly, a sample method based on stable populations such as HMRN could provide a cost-effective and reliable alternative to the current information strategy for haematological cancers.

## Appendix 1: Haematological Malignancy Research Network members

### Clinical network

Sam Ackroyd, Sara Ali, David Allsup, John Ashcroft, Rumana Bashi, Lee Bond, Geraldine Bynoe, David Bowen, Christopher Carter, Mary Chapple, Hanna Ciepluch, Gordon Cook, Ann Cuthbert, Jarek Czyz, Annette Edwards, Sylvia Feyler, Maria Gilleece, Di Gilson, Claire Hall, Emma Harris, Anita Hill, Quentin Hill, Pete Hillmen, Martin Howard, Sanjeev Jalihal, Rod Johnson, Sally Kinsey, Doris Kraemer, Michele Kwok-Williams, Tony Mcverry, Paul Moreton, Laura Munro, Lisa Newton, Derek Norfolk, Liaket Parapia, Kavita Patil, Russell Patmore, Mike Richards, Hazeem Sayala, Geoff Sherton, Graeme Smith, Kevin Speed, Andrew Steed, Chezhian Subash, Emma Thomas, Adrian Williams, Andrzej Wieczorek, David Wright.

### Haematological malignancy diagnostic service

Sharon Barrans, David Blythe, Cathy Burton, Matthew Cullen, Helen Dickinson, Paul Evans, Andrew Jack, Richard Jones, Sheila O'Connor, Roger Owen, Andy Rawstron, Steve Richards, David Swirsky, Reuben Tooze, Lisa Worrillow.

### Epidemiology and genetics unit

Pat Ansell, John Blase, Ian Cope, Helen Cox, Simon Crouch, Will Curson, James Doughty, Jan Farish, Susanne Griffiths, Debra Howell, Beki James, Tom Johnston, Eleanor Kane, Cath Lewis, Tracy Lightfoot, Rob Newton, Steven Oliver, Jane O'Sullivan, Dan Painter, Eve Roman, Jill Simpson, Vinnette Sinclair, Alex Smith, Anne Thomson.
